# Sport in ischemic heart disease: Focus on primary and secondary prevention

**DOI:** 10.1002/clc.24052

**Published:** 2023-05-28

**Authors:** Silvio Romano, Simona Minardi, Giampiero Patrizi, Zefferino Palamà, Alessandro Sciahbasi

**Affiliations:** ^1^ Cardiology, Department of Health, Life, and Environmental Sciences University of L'Aquila L'Aquila Italy; ^2^ Department of Cardiology B. Ramazzini Hospital, Ausl Modena Carpi Italy; ^3^ Interventional Cardiology Sandro Pertini Hospital Rome Italy

**Keywords:** cardiovascular prevention, ischemic heart disease, physical activity, sport

## Abstract

Ischemic heart disease (IHD) is one of the leading causes of death and morbidity in the world. The role of primary prevention is particularly relevant since IHD can be for a long time asymptomatic until the occurrence of a condition that could lead to plaque instabilization or increased oxygen demand. Secondary prevention is also essential to improve patients' prognosis and quality of life. The aim of this review is to provide a detailed and updated description of the role of sport and physical activity both in primary prevention and secondary prevention. In primary prevention, sport and physical activity are effective through the control of the main cardiovascular risk factors, such as hypertension and dyslipidemia. In secondary prevention, sport and physical activity can lead to a reduction in subsequent coronary events. Every effort must be made to encourage the performance of physical and sports activity both in asymptomatic subjects at risk and those with a history of IHD.

## 1 INTRODUCTION

Despite over the past few decades incidence and mortality rates of atherosclerotic cardiovascular disease are declining in many countries, there are currently still about 126 million cases of ischemic heart disease (IHD) in the world. IHD also remains one of the leading causes of death, accounting for over 9 million deaths per year worldwide.^1^, ^2^ The disease is most often progressive, and can have long stable periods but sometimes become unstable due to an acute atherothrombotic event or to oxygen supply/demand mismatch. Chronic coronary syndromes (CCS) and acute coronary syndromes (ACS) are two different manifestations of the same disease. CCS also displays several clinical scenarios^3^ such as: suspected IHD with “stable” symptoms, new onset of heart failure or left ventricular dysfunction in patients with suspected IHD, patients with stabilized symptoms <1 year after an ACS/revascularization, patients >1 year after initial diagnosis and, finally, asymptomatic subjects in whom IHD is detected at screening. Asymptomatic patients are those who most benefit from primary prevention. In fact, one of the main concerns is that CCS is often long‐time asymptomatic as, even in presence of significant atherosclerotic plaques, coronary blood flow at rest is frequently maintained by autoregulation processes, defined as the capacity to preserve blood flow during changes in perfusion pressure. Anyway, when the dilator reserve is compromised, the coronary blood flow becomes insufficient at high metabolic demands, for example, during physical exercise.^4^ Physical activity has undeniable benefits in the prevention of cardiovascular disease, therefore it must be advised to everyone, irrespective of the cardiovascular risk status, to maintain an optimal health condition. Moreover, in the presence of underlying CCS, intensive physical exercise may act as a trigger for life‐threatening ventricular arrhythmias and sudden cardiac death (SCD). Pre‐participation cardiovascular screening can be very important to early detect disorders associated with SCD, and to prescribe the safest exercise program for every single patient.

The purpose of this review is to analyse the current evidence on the beneficial role of sport and exercise in the primary and secondary prevention of IHD.

## 2 BENEFITS OF SPORT IN PRIMARY PREVENTION

The importance of physical activity for health and longevity has been promoted since ancient times. Even Hippocrates already advised that lack of physical exercise was detrimental to health.^5^ The benefits of physical activity in decreasing the likelihood of cardiovascular disease have been then well demonstrated. More recently, Manson et al., in 2002^6^ studied the role of walking and vigorous physical activities in the prevention of cardiovascular disease among 73 743 postmenopausal women 50−79 years old. During up to 5.9 years of follow‐up, even just walking was associated with substantial reductions in the incidence of cardiovascular events. The primary preventive impact of physical activity on all‐cause mortality and cardiovascular mortality was also assessed in a systematic review by Nocon et al.,^7^ including 33 cohort studies and 883 372 individuals. The follow‐up ranged from 4 years to over 20 years and the physical activity could be assessed by questionnaires or more objective measures of fitness. Most of the studies reported a significant reduction in coronary artery events, all‐cause mortality, and cardiovascular death for physically active people (risk reduction: 33% all‐cause mortality, 35% cardiovascular mortality). Studies that used only patient questionnaires to assess physical activity reported lower risk reductions.

Following papers confirmed the previous observations: Kokkinos et al.^8^ evaluated the cardio‐respiratory fitness (CRF) of 750 302 individuals (30−95 years old) based on peak METs achieved during a standardized exercise treadmill test. During a mean follow‐up of 10.2 years, the association of CRF and mortality risk was inverse, independent, and graded: the lowest mortality risk was observed at approximately 14.0 METs, in the absence of an increase in risk with extremely high CRF. Mortality risk for least fit individuals were fourfold higher compared with extremely fit individuals. Similar results had already been observed by Moore et al.^9^ with a pooled analysis, comprising six prospective cohort studies and 654 827 individuals, aimed at calculating the life expectancy years gained at different levels of leisure time activity. Physical exercise was categorized by metabolic equivalent hours per week (MET‐h/wk), while life expectancies and years of life gained/lost were calculated using directly adjusted survival curves. On a median follow‐up of 10 years, a physical activity level of 0.1−3.74 MET‐h/wk, equivalent to brisk walking for up to 75 min/wk, was associated with a gain of 1.8 years in life expectancy versus no leisure time activity (0 MET‐h/wk). Higher levels of physical activity were associated with greater gains in life expectancy (on the order of 5 years, if a good level of physical activity was reached).

Different mechanisms by which physical activity may prevent cardiovascular disease have been assumed, such as changes in blood pressure (BP), vascular function, insulin sensitivity, lipids pathway, skeletal muscle metabolism, and body mass index.^10^ On the other hand, it was also shown that physical exercise might prevent cardiovascular diseases independently of its potential benefit on cardiovascular risk factors: some changes in inflammatory and hemostatic activation (reduced blood concentrations of several inflammatory biomarkers such as C‐reactive protein, lipoprotein‐associated phosphor‐lipase A2, cytokines interleukin [IL]‐1β, IL‐6, and tumor‐necrosis factor‐α) have been observed in athlete's laboratory analysis and were supposed to be possible underlying factors.^11^ Furthermore, even when exercise programs in primary prevention fail to prevent people from ACS, it seems to act in a protective way as well. An interesting analysis by Pitsavos et al.^12^ specifically evaluated the association between physical activity levels and clinical outcomes at presentation and 30‐day prognosis of hospitalized patients with ACS. An inverse association was observed between the level of physical activity and troponin‐I at presentation; furthermore, prior physical activity was associated with reduced severity of ACS, reduced in‐hospital mortality rates, and improved short‐term prognosis.

Since physical activity beneficially influences most of the atherosclerotic risk factors and preserves the maintenance of good CRF, current guidelines recommend at least 150 min/per wk of moderate‐intensity, or 75 min/per wk of vigorous‐intensity aerobic exercise in all healthy adults.^13^

### 2.1 Hypertension and sport

Hypertension, considered as persistent systolic blood pressure (SBP) ≥140 mmHg and/or diastolic blood pressure ≥90 mmHg, is a very common condition, especially among people over 35 years. Strong evidence indicates a role of moderate to vigorous physical activity in preventing hypertension, suggesting an inverse relationship between exercise and incident hypertension among normotensive and prehypertensive individuals.^14^ The earliest study to demonstrate the protective effects of physical activity was published in 1968 by Paffenbarger et al.^15^ who showed that men who exercised more than 5 h/wk experienced a lower incidence of hypertension and two to three decades later in life.

Regular physical activity has also an antihypertensive function. Börjesson et al.^16^ analyzed the BP lowering effect of aerobic physical activity in 27 randomized controlled studies on individuals with hypertension: regular medium‐to‐high‐intensity aerobic activity reduced the BP by a mean of 5/11 mmHg. Indeed, hypertensive people are nowadays recommended to practice at least 30 min of moderate‐intense dynamic aerobic exercise (walking, jogging, cycling, or swimming) for 5−7 days per week.^17^

People who suffer from hypertension and want to take part in sports activities, should be properly evaluated as regard their risk profile and the activity intensity they'd like to perform. Current guidelines^13^, ^18^ suggest four risk levels (low, moderate, high, and very high) according to the hypertension grade and the presence of other risk factors or disease. Once established the risk, the recommendations are different: (i) hypertensive individuals at high and very high cardiovascular risk should be only eligible to skills sports; (ii) hypertensive individuals at moderate cardiovascular risk, once demonstrated well‐controlled BP during exercise (SBP during exercise <240 mmHg for men and <220 mmHg for women) may be eligible to competitive sports except for activities requiring strenuous efforts, even for a short time (weightlifting, bodybuilding); (iii) hypertensive individuals at low cardiovascular risk may be eligible to every type of competitive sport; anyway, if high‐intensity sports participation is desired, a pre‐participation assessment to identify athletes with exercise‐induced symptoms and excessive BP response to exercise is warranted. All these efforts are aimed at ensuring the performance of sporting activities in safety.

#### 2.1.1 Management of hypertension in athletes

Hypertension management in athletes requires a step‐by‐step approach. The first step is a nonpharmacological strategy: salt restriction, weight reduction if obesity is present, alcohol restriction, strict nicotine cessation, healthier eating habits, and discontinuation of anti‐inflammatory therapy. If the lifestyle changes fail to lower the BP after 3 months, antihypertensive medications should be considered.^18^ The choice of an anti‐hypertensive drugs for athletes requires particular attention. The ideal drug should not decrease the cardiac response to exercise, not be pro‐arrhythmic, ensure normal blood distribution to the working muscles, do not interfere with the normal use of energy substrate. Analyzing the effects of principal antihypertensive medications on athlete's performance^19^: ACE‐Inhibitors and ARBs have little to no effect on exercise capacity and minimal effects on heat illness threshold; on the other hand, they may fail to control hypertension in black athletes as well, are contraindicated in women of childbearing potential, are linked to a relatively high incidence of angioedema (higher in blacks and Latinos/Latinas) and requires to serum creatinine and K+ checks 1−2 weeks after initiation/dose change. Dihydropyridine Calcium Channel Blockers do not significantly affect heart rate (HR), have a small negative effect on VO_2max_, are particularly useful in black athletes and no routine laboratory monitoring is required; on the other hand, they decrease heat illness threshold and some dose‐dependent peripheral edema are reported. Beta‐blockers are poorly tolerated due to their adverse effects on exercise capacity and their use is banned by the World Anti‐Doping Agency (WADA) for people performing sports requiring fine motor movements. Diuretics are banned by most sports oversight committees since they increase the risk of dehydration, decrease the heat illness threshold and may precipitate participation and post‐participation muscle cramps. Moreover, they are considered masking agents by WADA.

### 2.2 Dyslipidaemia and sport

One of the mechanisms proposed to explain the association between physical activity and decreased IHD incidence is the beneficial effect of exercise on lipid profile.^20^ Important benefits of physical activity on lipidic metabolism include positive impacts on plasma Triglycerides (TG), high‐density lipoprotein (HDL), low‐density lipoprotein (LDL), and total cholesterol (TC).^21^, ^22^ Already in 1983 Tran et al.,^22^ in a meta‐nalysis including 66 studies and a total of 2925 patients, studied the effects of physical activity on blood lipids and lipoproteins. They observed a reduction in TC, TG, LDL, and an increase in HDL among exercising individuals. Following studies confirmed these findings: Kokkinos et al.^23^ examined the association between miles run per week and HDL levels in 2906 healthy middle‐aged men. The results indicated that with increasing miles run per week, not only gradually raised the HDL levels, but also improved the levels of LDL, TG, and TC/HDL ratio. In a recent cohort study Momma et al.^24^ examined the association of grip strength and other physical fitness markers with the incidence of dyslipidemia among 16 149 Japanese adults without a history of cardiovascular disease or dyslipidemia at the basal time, showing that a higher relative grip strength was associated with a lower incidence of dyslipidemia among both men and women on a follow‐up of 4.5 years.

The components of lipids that are most amenable to change with exercise implementation are TG and HDL In particular the enzyme lipoprotein lipase that is critical in the formation of HDL is increased with aerobic exercise.^25^ A large cross‐sectional study by Da Silva et al.^26^ assessed the association of physical activity with HDL, LDL, and TG on 12 688 individuals who were not on lipid‐lowering medication: moderate and vigorous physical activity increased mean HDL level by 0.89 and 1.71 mg/dL, respectively, and reduced TG geometric mean by 0.98 and 0.93 mg/dL, respectively. Vigorous physical activity was also associated with lower LDL. Many studies and metanalysis focused their attention on plasma TG: Lampman et al.^27^ showed that the effects of exercise on subjects with mild to moderate elevated TG levels were more marked and longer‐lasting when associated with diet, while other Authors observed a significant reduction in TG despite the calories dietary intake^28^ and independently from the weight loss.^29^ A more recent systematic review by Jimenez et al.,^30^ including 36 studies, evaluated the comparative effectiveness of statins versus aerobic exercise in reducing fasting and postprandial TG (PPTG) concentrations in individuals at high risk of developing IHD. The results showed that statin and exercise interventions carried similar reductions in PPTG levels. Evidence regarding the effect of exercise on LDL is conflicting: most studies have shown favorable effects,^23^, ^24^, ^26^ especially when high‐amount–high‐intensity exercise was performed,^31^ while some other papers did not observe similar results.^32^, ^33^

#### 2.2.1 Management of dyslipidemia in athletes

The 2013 AHA/ACC Lifestyle Management Guidelines to Reduce Cardiovascular Risk^34^ recommend 3−4 sessions per week, lasting on average 40 min per session, and involving moderate‐ to vigorous intensity physical activity to reduce LDL and non‐HDL cholesterol. This recommendation is mainly confirmed by the recent 2021 ESC guidelines on cardiovascular disease prevention in clinical practice.^35^ Anyway, despite the benefits of physical exercise on lipidic profile, dyslipidemia is one of the most common risk factors among athletes^36^ and often requires to be managed as well as in the general population. Once excluded possible secondary causes, such as thyroid disorders, familial hypercholesterolemia, and side effects of anabolic steroids, the first step in the general population is lifestyle interventions. Sportsmen generally follow healthy lifestyles even if some athletes prefer weight gain with the goal of becoming larger than their opponents (es. rugby players, soccer players, swimmers, etc). Therefore, drug treatment may be required and among the possible treatments, statins are the first line. Statin use in athletes is limited by the reported increase of myopathy. Aching, cramps, and muscle weakness increases with strenuous exercise and are frequent causes of statins discontinuation in the general population as well as among athletes. Sinzinger et al.^37^ monitored 22 professional athletes in whom, because of familial hypercholesterolemia, treatment with different statins was attempted. They found that muscular problems during exercise were the major side‐effect during statin treatment, occurring more frequently during or after exercise, even in the absence of elevated creatine kinase. In this population of top sports performers only about 20% tolerated statin treatment without side‐effects. Factors that may predispose athletes to statins intolerance are the type of statin, dose, drug interactions, genetic variants, coenzyme Q10 deficiency, vitamin D deficiency, and underlying muscle diseases.^38^ Some strategies to decrease the risk of adverse interactions between statins and exercise were suggested: frequently reassessing the need for statin and reducing the dose when possible, changing to a hydrophilic statin, prescribing a “statin holiday” followed by a rechallenge, prescribing replacement of vitamin D and supplementation of coenzyme Q10 and l‐carnitine and avoiding drug interactions that increase statin toxicity.^38^ Moreover, today we also have other available resources that can be used in combination or as an alternative to statin therapy in case of intolerance. These tools comprehend: ezetimibe, nutraceuticals, bempedoic acid, PCSK9‐inhibitors, and SiRNA.

An‐depth analysis of single substances is beyond the scope of this review.

## 3 THE ROLE OF PRE‐PARTICIPATION SCREENING IN PRIMARY PREVENTION

Pre‐participation screening is important to prevent SCD both in young and master athletes. In Italy, a precise protocol, standardized by a State law since 1982,^39^ requires mandatory investigation such as clinical evaluation (physical examination and medical history), ECG, stress test, and yearly re‐evaluation. The efficacy of Italian experience (based on rest and stress ECG) is clearly demonstrated by Corrado et al.^40^ who showed a significant decrease in annual rates of SCD in screened competitive athletes versus unscreened nonathletes aged 12−35 years. In these young individuals, SCD is mainly due to diseases other than IHD (hypertrophic, arrhythmogenic and dilated cardiomyopathies, congenital coronary artery disease, and ion channelopathies) while IHD is the commonest cause of sport‐related SCD among people older than 35. In this latter age group, the role of pre‐participation screening in IHD primary prevention may be particularly relevant as the population of master athletes is numerically increasing. Even if pre‐participation evaluation includes medical history, physical examination, ECG, and stress test, the initial risk stratification is also very important, at least evaluating glycaemic and cholesterol levels, BP, and smoking anamnesis. However, routine screening for ischemia with exercise testing in asymptomatic adults generally has a low positive predictive value. Thus, treadmill or cycle ergometer maximal exercise test should be interpreted with a poli‐parametric analysis, not only looking at ST deviation but globally evaluating: exercise capacity (time of exercise and METs), HR (chronotropic competence, HR postexercise recovery), BP response, hypertensive or hypotensive, abnormal BP recovery, supraventricular or ventricular arrhythmias, angina development, pharmacological therapy (response/modifications). In adult and elderly individuals, especially those who want to begin with moderate to vigorous physical activities, cardiopulmonary exercise testing may be a useful tool to assess overall CV health and performance, allowing individualized recommendations regarding sports and exercise type and intensity.^41^ Additional tests should be requested for athletes who had positive/doubt findings at the initial evaluation (i.e., coronary CT scan and stress imaging exams).

## 4 BENEFITS OF SPORT IN SECONDARY PREVENTION

Physical activity, in addition to medical therapy, has been demonstrated to have significant benefits in the secondary prevention of IHD. A recent systematic review and metanalysis by Salzwedel et al.,^42^ including 31 studies and 228 337 patients, confirmed the effectiveness of cardiac rehabilitation participation even after an ACS and after coronary artery bypass grafting reducing total mortality in addition to the current evidence‐based coronary artery disease treatment. Benefits in terms of all‐cause mortality and CV death were also recently observed by Gonzales‐Jeramillo et al.^43^ in a prospective cohort study that included 33 576 patients affected by coronary heart disease. Physical activity was assessed through validated questionnaires and changes in activity level was recorded. Compared with always‐inactive patients, the risk of all‐cause mortality was 50% lower in those who were active and remained active, 45% lower in those who were inactive but became active, and 20% lower also in those who were active but became inactive.

The mechanism by which regular physical exercise reduces cardiovascular mortality and morbidity in IHD patients may rely, as well as in the general population, on changes in body fat percentage, lipoprotein profile, carbohydrate tolerance and insulin sensitivity, neurohormonal release, and BP. Furthermore, exercise appears to modify the vascular structure, endothelial function and microvasculature, improving myocardial perfusion in IHD patients.^44^ Indeed, the shear stress that occurs during physical activity seems to improve endothelial function, stimulating endothelial production of nitric oxygen and antioxidant substances.^45^ The effect of physical exercise on IHD patients is also demonstrated by studies on symptoms and coronary disease progression. Schuler et al.^46^ conducted an interventional study on 36 patients affected by symptomatic IHD observing that regular controlled physical exercise at high intensity and normalization of serum lipoproteins improved myocardial perfusion assessed with 201‐Thallium scintigraphy at 1‐year follow‐up. Neubauer et al.^47^ looked at the long‐term effects of physical exercise and a low‐fat diet on the progression of coronary artery disease assessed by coronary angiography: interventional group patients showed significant delay of disease progression at 6 years. Hambrecht et al.^48^ evaluated the effect of different levels of physical activity progression of coronary atherosclerotic lesions in patients with IHD: higher workloads were needed to stop the progression of coronary atherosclerotic lesions (1533 ± 122 Kcal/wk), but regression of coronary lesions was only observed for an average of 2200 kcal/wk, amounting to 6 h/wk of regular physical exercise.

Therefore, exercise training is a major component of IHD secondary prevention and cardiac rehabilitation, to attenuate disease progression and improve patients' prognosis.

## 5 EXERCISE PRESCRIPTION

Exercise prescription commonly refers to fitness‐related activities that are designed for a specified purpose, often prepared for the individual by a fitness or rehabilitation specialist.^49^ Exercise is safe and effective for most the subjects; however, all individuals should be stratified on their risk for the occurrence of a cardiac‐related event during exercise training. In particular, an accurate evaluation is required for exercise prescription both in primary prevention for high cardiovascular risk patients, asymptomatic patients in which IHD is detected during screening tests and in secondary prevention for patients who underwent myocardial infarction or coronary revascularization. Prescriptive techniques for determining exercise dosage must be based on personalized programs according to the accurate quantification of the patient's exercise capacity. The right prescription is driven by four principal pillars, following the “FITT” model^41^, ^50^: Frequency, Intensity, Time, Type.

Frequency is how often a person does the exercise activity; Intensity is how hard a person works to do the activity; Time is how long a person does an activity in any one session; Type is the kind of physical activity it could be aerobic, muscle‐strengthening, bone‐strengthening, and flexibility activities. How much exercise to prescribe in terms of “FITT” depends on the individual evaluation that should include medical history, physical examination, HR, BP, body weight, ECG, and stress test. Symptoms or evidence of change in clinical status, not necessarily related to activity, as well as evidence of exercise intolerance, must also be investigated and, when required, further diagnostic tests could be considered. *Frequency* depends on several factors including baseline exercise tolerance, its intensity, individual fitness status, and types of exercise incorporated into the program. The recommendations for individuals with cardiovascular disease^50^ suggest at least 3 training sections per week, preferably 5 if aerobic activities, or 2−3 nonconsecutive if resistance activities or ≥2−3 if flexibility activities are performed. According to *time*, it is recommended 20−60 min of aerobic activities or 1−3 sets of resistance activities, comprehending 8−10 different exercises focused on major muscle groups, or 15 s hold for static stretching with ≥4 repetitions of each flexibility exercise.^50^ The most recommended *type* of exercise for individuals with CVD is aerobic physical activity, which consists of rhythmic movements involving large muscle groups for an extended period with an emphasis on increased caloric expenditure for the maintenance of healthy body weight. Aerobic training sessions are the most effective in improving cardiorespiratory fitness. Anyway, the key point in prescribing exercise is to establish its appropriate *intensity*. The exercise intensity depends on the energy expended during the effort and can be evaluated in different ways. A direct expression of intensity is the METS or the expended kcals; an indirect expression is by means of energy expenditure indices that can be quantified through an exercise test or expressed in a qualitative way by the use of subjective parameters (i.e., the Borg scale or the Talk Test^41^). Actual guidelines^50^ recommend aerobic activities at the intensity of 40%−80% of exercise capacity. There are several methods to determine this percentage, including or not an exercise test and characterized by different accuracy. The most accurate tool to evaluate cardiovascular response to a specific intensity of exercise is the Cardio‐Pulmonary Exercise Test, recommended for patients at higher CV risk, to establish the HR reserve, oxygen uptake reserve (VO_2_R), or peak oxygen uptake (VO_2_peak) thresholds. The main variable of maximal oxygen uptake (VO_2max_) reflects the gold standard measure of exercise capacity and, together with information derived by cardiac frequency and subjective symptoms, it contributes to defining the safest training program for the individual patient.^41^, ^50^ Alternatively, if an exercise test is not performed, easier methods to establish the individual exercise capacity could be considering +20/+30 beats/min over the HR resting (HR rest) or a rating of perceived exertion of 12−16 on a scale of 6−20.

An adequate assessment of the type and intensity of physical exercise to be prescribed is also very important to ensure safe physical activity. In this regard, Hauer et al.^51^ assessed whether exercise‐induced myocardial ischemia during intensive group exercise sessions in patients with coronary artery disease and stable angina pectoris could be predicted by routine diagnostic procedures, including coronary angiography, symptom‐limited exercise testing with 201‐Thallium scintigraphy and simultaneous ECG recording. The results showed that routine diagnostic exams do not sufficiently identify patients at risk for exercise‐induced myocardial ischemia, since a higher number of patients suffered from myocardial ischemia during daily sports activities rather than during exercise tests. Interestingly, all the ischemic events occurred in patients who exceeded the recommended HR thresholds and were only effectively prevented by choosing adequate types of exercise and, above all, by the strict adherence to individual target HR. Therefore, the training schedule should be adapted according to the patient's disease severity, frailty, and baseline exercise capacity.

## 6 CONCLUSIONS

Physical and sport activity has a crucial role in the primary and secondary prevention of IHD. Every effort must be made to encourage the performance of physical and sports activity both in asymptomatic subjects at risk and those with a history of IHD. Furthermore, particular attention must be paid to prescribing the right amount of physical exercise for each subject, in the logic of precision medicine.

## Data Availability

Data sharing is not applicable to this article as no new data were created or analyzed in this study.
